# Effect of Urinary Bisphenol A on Androgenic Hormones and Insulin Resistance in Preadolescent Girls: A Pilot Study from the Ewha Birth & Growth Cohort

**DOI:** 10.3390/ijerph10115737

**Published:** 2013-11-01

**Authors:** Hye Ah Lee, Young Ju Kim, Hwayoung Lee, Hye Sun Gwak, Eun Ae Park, Su Jin Cho, Hae Soon Kim, Eun Hee Ha, Hyesook Park

**Affiliations:** 1 Department of Preventive Medicine, School of Medicine, Ewha Womans University, Seoul 158-710, Korea; E-Mails: khyeah@naver.com (H.A.L.); eunheeha@ewha.ac.kr (E.H.H.); 2 Department of Obstetrics and Gynecology, School of Medicine, Ewha Womans University, Seoul 158-710, Korea; E-Mail: kkyj@ewha.ac.kr; 3 Department of Anatomy, School of Medicine, Ewha Womans University, Seoul 158-710, Korea; E-Mail: hylee38@ewha.ac.kr; 4 Colleage of Pharmacy, Ewha Womans University, Seoul 120-750, Korea; E-Mail: hsgwak@ewha.ac.kr; 5 Department of Pediatrics, School of Medicine, Ewha Womans University, Seoul 158-710, Korea; E-Mails: pea8639@ewha.ac.kr (E.A.P.); sujin-cho@ewha.ac.kr (S.J.C.); hyesk@ewha.ac.kr (H.S.K.)

**Keywords:** androgenic hormones, bisphenol A, child health, endocrine disruptor chemicals, insulin resistance

## Abstract

To assess the effect of urinary bisphenol A (BPA) on repeated measurements of androgenic hormones and metabolic indices, we used multivariate analysis of variance (MANOVA) adjusted for potential confounders at baseline. During July to August 2011, 80 preadolescent girls enrolled in the Ewha Birth & Growth Cohort study participated in a follow-up study and then forty-eight of them (60.0%) came back one year later. Baseline levels of estradiol and androstenedione were higher in the BPA group than in the non-BPA group. One year later, girls in the high BPA exposure group showed higher levels of androstenedione, testosterone, estradiol, and insulin, and homeostasis model assessment of insulin resistance (HOMA-IR) index, than those in the other groups (*p* < 0.05). In MANOVA, estradiol and androstenedione showed significant differences among groups, while dehydroepiandrosterone, insulin, and HOMA-IR showed marginally significant differences. Exposure to BPA may affect endocrine metabolism in preadolescents. However, further investigation is required to elucidate the mechanisms linking BPA with regulation of androgenic hormones.

## 1. Introduction

With the decline in birth rate from 13.3 per 1,000 people in 2000 to 9.4 per 1,000 people in 2011 [1] and the increases in infertility and mean age of women at first birth [2], reproductive health is becoming a major public health challenge in the Republic of Korea. Accumulating evidence from animal studies suggests that some environmental chemicals potentially cause a wide range of reproductive abnormalities [3]. Bisphenol A (BPA), an endocrine-disrupting chemical (EDC), is widely used today as a plasticizer and stabilizer in the manufacture of consumer products [4]. It is well known for its estrogen-mimicking properties, and was recently suggested to also play a role in androgen metabolism [5]. In the past few decades, several *in vivo* and *in vitro* studies have implied the existence of a bidirectional interaction between BPA and androgenic hormones [6,7,8,9]. However, the scientific mechanisms of BPA remain unclear. In addition, human data supporting androgen-related activities of BPA are lacking.

In a recent case-control study, serum BPA concentrations were found to be significantly higher in patients with polycystic ovary syndrome (PCOS) compared to controls (1.05 ± 0.56 *vs*. 0.72 ± 0.37 ng/mL, *p* < 0.001) [7]. PCOS is the most common endocrinopathy in women of reproductive age. It is characterized by symptoms such as hyperandrogenemia, insulin resistance, and chronic anovulation [10]. Ropero *et al*. reported that BPA-mediated disruption of pancreatic β-cell function could induce insulin resistance [11]. The National Health and Nutrition Examination Survey 2003–2004 reported that higher BPA concentrations were associated with diabetes [12].

Children are a vulnerable group in terms of endocrine-disrupting chemical exposure. Calafat *et al*. [13] reported that younger children aged 6–11 years had a higher BPA concentration compared to older age groups. This is explained by the higher levels of food consumption in this age group. However, evidence is lacking on the effect of BPA exposure on endocrine health, particularly in subjects who have not yet acquired reproductive ability.

We assumed that a single-spot urinary BPA concentration would represent chronic exposure and hypothesized that this exposure would persistently affect endocrine activity, notably the androgenic hormones testosterone, androstenedione, and dehydroepiandrosterone (DHEA), as well as insulin resistance, among girls of pre-reproductive ages. By measuring urinary BPA concentrations of girls from the Ewha Birth & Growth Cohort who were aged 7 to 8 years (*i.e.*, before the onset of menarche), we investigated the effect of BPA on androgenic hormones and insulin resistance during a 1-year follow-up period.

## 2. Materials and Methods

### 2.1. Study Population

The study subjects were from the Ewha Birth & Growth Cohort study, which is an ongoing longitudinal birth cohort study established at Mokdong Hospital, Ewha Woman’s University, Seoul, South Korea between 2001 and 2006. Details of cohort composition and methodology are described in a previous study [14]. The follow-up program of the Ewha Birth & Growth Cohort study has been conducted annually since 2005, when subjects had reached 3, 5, 7, 8, and 9 years of age. During July to August 2011, we conducted a follow-up examination of children aged 7 to 8 years. Further follow-up examinations were performed 1 year later. At the time of follow-up, we informed parents or guardians about the follow-up program by telephone and subjects then visited the hospital. Those who participated in the follow-up provided detailed data through a questionnaire and blood and urine samples. To minimize bias while processing, we performed the follow-up program in the morning at an interval of ~7 days during the same month each year. All participants fasted for at least 8–12 h before specimen collection (blood and urine). Venous blood was collected from the antecubital vein, and urine samples were collected in the morning at the hospital using polypropylene urine cups and were stored at −80 °C until analysis. All subjects and parents gave their written informed consent to participate in this study.

To meet the study objectives, the present study was limited only to girls. Of the 155 subjects who participated in 2011, 80 (51.6%) were girls, 48 of whom were examined twice. Comparisons of baseline characteristics between subjects who participated annually in the follow-up examination (n = 48) and those who participated once (n = 32) are summarized in Supplemental Data 1. Among females in the Ewha Birth & Growth Cohort, there were no differences in maternal age at delivery or birth outcomes between participants (n = 80) and non-participants (n = 123) in the 2011 follow-up. The characteristics (participants *vs*. non-participants, respectively) were as follows: maternal age at birth = 31.71 ± 3.35 *vs*. 30.98 ± 3.55 years, *p* = 0.15; birth weight = 3.21 ± 0.04 *vs*. 3.16 ± 0.04 kg, *p* = 0.76; gestational age = 39.2 ± 1.7 *vs*. 39.1 ± 1.6 years, *p* = 0.35. The study protocol was approved by the Institutional Review Board of Ewha Woman’s University Hospital.

### 2.2. Urinary BPA Measurements

Urinary BPA concentrations in the subjects were determined by high-performance liquid chromatography with fluorescence detection (LC-10SPD10-AVvp system, Shimadzu, Tokyo, Japan). A total of 30 µL 2.0 M sodium acetate (pH 5.0) and 10 µL β-glucuronidase was added to a 1 mL urine sample, which then was incubated for 3 h at 37 °C. After incubation, 100 μL 2 M HCL was added. Standard bisphenol B (50 ng/mL) was mixed with 4 mL ethyl acetate and then extracted. After 3 mL supernatant was dried, it was dissolved in 70 μL 60% acetonitrile, and then analyzed using a 5 µm column (4.60 × 150 mm) (Luna 5u C18). The mobile phase consisted of mixture of 2.5% tetrahydrofuran/acetonitrile buffer (70:30) and was delivered at a flow rate of 1.5 mL/min. Fluorescence detection was performed at an excitation wavelength of 275 nm and an emission wavelength of 300 nm. The limit of detection (LOD) was 2.0 ng/mL. A linear calibration was obtained from 2.5–100 ng/mL (R^2^ > 99.9%). Statistical analyses of the data included urinary BPA measurements below the LOD. BPA concentrations below the LOD (17.5%) were assigned a concentration of LOD/2. We adjusted for urinary creatinine in multivariate analysis of variance and repeated measures. To compare exposures among groups, subjects were classified into tertiles based on urinary BPA concentrations at baseline (tertile 1: <5.36, tertile 2: 5.36~12.38, and tertile 3: 12.39~25.31 ng/mL).

### 2.3. Anthropometric Measurements and Pubertal Development Assessment

Anthropometric data were collected by well-trained researchers when subjects visited the hospital. Height, waist circumference, and weight were measured to the nearest 0.1 cm or 0.1 kg using a stadiometer and calibrated scale (DS-102, Dong Sahn Jenix Co. Ltd., Seoul, Korea) with the children wearing light clothing but no shoes. Body mass index (BMI) (kg/m^2^) was calculated as weight divided by height squared. Height, weight, and BMI were transferred to an age- and gender-specific z score criteria reference source from the 2007 Korean Children and Adolescents Growth Standards [15]. Pubertal development was determined based on clinician-reported Tanner stage assessments. We categorized Tanner stage 2 or over as onset of puberty.

### 2.4. Sex Hormone and Metabolic Indices Measurements

Subjects were instructed to fast for at least 8 h prior to giving a blood sample. All blood samples obtained from the subjects were stored at −80 °C until analysis. Luteinizing hormone (LH) (KA0214; ABNOVA, Taipei City, Taiwan), DHEA (IMaa38; IMMUNOTECH, Marseille, France), androstenedione (KIP0451; DIAsource ImmunoAssays, S.A., Louvain-la-Neuve, Belgium), and free testosterone (KIP1709; DIAsource ImmunoAssays S.A.) were measured using a commercial kit, according to the manufacturer’s protocols. Free estradiol concentrations in serum were measured by a competitive electrochemiluminescence immunoassay method using an Elecsys E170 analyzer (Elecsys Estradiol II; Roche Diagnostics GmbH, Mannheim, Germany). Serum insulin was measured using an immunoradiometric assay kit (Biosource Europe, Nivelles, Belgium). Glucose concentrations were measured with an automatic analyzer (model 7180; Hitachi, Tokyo, Japan). Insulin resistance was determined by the commonly used index homeostasis model assessment of insulin resistance (HOMA-IR), calculated as (plasma glucose (mmol/L) × insulin (μIU/mL))/22.5. All measurements were made under conditions with inter- and intra-assay coefficient of variation (CV) values of less than 10% (Table 1).

**Table 1:** Precision and sensitivity of measurements.

	Sensitivity	Inter-assay of coefficient variation	Intra-assay of coefficient variation
LH (IU/L)	0.25 IU/L	5.7%–8.1%	5.8%–6.8%
Estradiol (pg/mL)	5.00 pg/mL	2.2%–4.7%	1.7%–3.3%
Androstenedione (ng/mL)	0.03 ng/mL	5.9%–9.0%	3.2%–4.5%
DHEA (ng/mL)	0.13 ng/mL	10.4%–11.9%	6.9%–7.9%
Testosterone (ng/mL)	0.02 ng/mL	4.8%–6.2%	3.3%–4.6%
Insulin (μIU/mL)	0.20 μIU/mL	2.4%–4.9%	0.8%–1.5%
Glucose (mmol/L)	0.11 mmol/L	0.81%–1.12%	<5.0%

### 2.5. Statistical Analysis

Analysis of variance (ANOVA) was used to compare urinary BPA tertiles in terms of the following continuous measures: height, weight, BMI, age- and gender-specific z scores for the three previous parameters, waist circumference, insulin, glucose, and HOMA-IR. Differences in sex hormone levels (LH, estradiol, androstenedione, DHEA, and testosterone) according to BPA groups were evaluated by a Kruskal-Wallis test. Distributions of categorical variables according to urinary BPA tertiles were assessed by chi-squared test and Fisher’s exact test if more than 20% of the cells had expected values of <5. The Cochran-Mantel-Haenszel test was used to assess the trend. The baseline characteristics of all participants and non-participants were compared using the *t*-test and Mann-Whitney U test.

Potential confounding factors at baseline were selected using data from the literature [16] and the results of descriptive analysis (*p* < 0.05). To assess the effect of urinary BPA concentration on repeated measurements of androgenic hormones and metabolic indices, we used multivariate analysis of variance (MANOVA) with adjustments for age, household income, urinary creatinine level, and pubertal level (measured by Tanner stage at baseline). Prior to analysis, sex hormones were log transformed to satisfy statistical normality. The results are presented as the exponential value of the log scale. Multiple comparisons of BPA tertiles were conducted using the Tukey adjustment method. All analyses were two-tailed and were conducted using SAS software (ver. 9.1; SAS Institute, Cary, NC, USA). A *p*-value < 0.05 was considered to be statistically significant. 

## 3. Results

Total BPA was measured in 82.5% of the spot-urine samples, with a median concentration of 12.0 ng/mL (inter-quartile range (IQR): 4.0–20.7 ng/mL). The median creatinine-adjusted concentrations were 1.97 μg/g creatinine (tertile 1 IQR: 0.90–3.93), 10.79 μg/g creatinine (tertile 2 IQR: 8.06–18.38), and 22.51 μg/g creatinine (tertile 3 IQR: 15.06–44.83). For repeated measures of BPA concentration, the Spearman correlation was 0.36 (*p* < 0.02) between T1 and T2 for 48 subjects.

The relationship between BPA concentration tertile and BMI demonstrated an U-shape, but was not statistically significant. Girls in the top BPA exposure tertile were more likely to have reached Tanner stage 2 or more (7.4% in BPA tertile 1, 14.8% in BPA tertile 2, and 15.4% in BPA tertile 3), although not significantly so. The proportion of children with a high household income at baseline was significantly increased for subjects with a higher BPA tertile (25.9% in BPA tertile 1, 40.7% in BPA tertile 2, and 50.0% in BPA tertile 3; *p* for trend < 0.05). However, parental education status did not differ according to BPA group (Table 2). Repeated measurements of endocrine hormone levels were significantly correlated with each other, except for those for LH (*r* = 0.23, *p* > 0.05 for LH; *r* = 0.73, *p* < 0.0001 for testosterone; *r* = 0.67, *p* < 0.0001 for androstenedione; *r* = 0.49, *p* < 0.001 for estradiol, *r* = 0.59, *p* < 0.0001 for DHEA; *r* = 0.37, *p* = 0.01 for insulin; *r* = 0.51, *p* < 0.001 for glucose; and *r* = 0.39, *p* < 0.01 for HOMA-IR).

**Table 2:** Baseline characteristics of study subjects based on urinary bisphenol A concentration (n = 80).

	Total	BPA			*p*
		Tertile 1 (n = 27)	Tertile 2 (n = 27)	Tertile 3 (n = 26)	
Age	7.45 (0.50)	7.52 (0.51)	7.37 (0.49)	7.46 (0.51)	0.55
Height	126.16 (5.82)	125.63 (5.48)	125.90 (5.06)	126.98 (6.94)	0.68
Height z score	0.35 (0.88)	0.19 (0.97)	0.39 (0.72)	0.49 (0.93)	0.44
Weight	25.77 (5.38)	25.62 (4.69)	24.67 (4.06)	27.07 (6.96)	0.27
Weight z score	0.00 (0.98)	−0.05 (0.95)	−0.14 (0.98)	0.21 (1.02)	0.40
BMI	16.07 (2.30)	16.13 (2.02)	15.51 (1.91)	16.59 (2.84)	0.23
BMI z score	−0.27 (1.10)	−0.20 (0.94)	−0.53 (1.20)	−0.06 (1.14)	0.28
Waist circumference (cm)	54.82 (6.36)	54.34 (3.94)	54.17 (4.93)	56.00 (9.18)	0.52
Tanner’s stage (2 or more), n (%)	10 (12.35%)	2 (7.41%)	4 (14.81%)	4 (15.38%)	0.70 ^†^
Paternal education level, n (%)					
≤high school	8 (10.00%)	3 (11.11%)	4 (14.81%)	1 (3.85%)	0.52
Above college	72 (90.00%)	24 (88.89%)	23 (85.19%)	25 (96.15%)	
Maternal education level, n (%)					
≤high school	16 (20.00%)	4 (14.81%)	8 (29.63%)	4 (15.38%)	0.31
Above college	64 (80.00%)	23 (85.19%)	19 (70.37%)	22 (84.62%)	
Household income, n (%)					
<3 million KRW	12 (15.00%)	6 (22.22%)	5 (18.52%)	1 (3.85%)	0.20 ^‡^
3~4.9 million KRW	37 (46.25%)	14 (51.85%)	11 (40.74%)	12 (46.15%)	
≥5 million KRW	31 (38.75%)	7 (25.93%)	11 (40.74%)	13 (50.00%)	

Data are presented as the mean plus standard deviation for continuous variables and number (%) for categorical and ordinal variables; BPA, bisphenol A; BMI, body mass index; KRW, Korean won; ^†^ Fisher’s exact test; ^‡^ Mantel-Haenszel test = 0.03.

Table 3 summarizes the association between urinary BPA exposure group and sexual hormone levels at each time point. At baseline (time 1, T1), girls in the top tertile for urinary BPA had the highest values for almost all parameters, but there were no significant differences. At 1 year later (time 2, T2), there were significant differences for estradiol, androstenedione, testosterone, insulin, and HOMA-IR (Tukey-adjusted *p* < 0.05).

In MANOVA, there were significant differences among BPA exposure groups for androstenedione, estradiol, insulin, glucose, and HOMA-IR, and marginally significant differences for DHEA and testosterone (data not shown), after adjustments for age, household income, and Tanner stage. However, the differences for insulin, glucose, and HOMA-IR were attenuated after further adjustment for urinary creatinine level; DHEA retained a marginal association (Table 4). In girls in the high BPA exposure group the changes from baseline in least squares means for estradiol and DHEA were high. In addition, the concentrations of androstenedione in girls in the top tertile increased over time (difference [d] = 0.04). Similar trends were not observed in the other groups (d for the intermediate tertile = −0.01, d for the lowest tertile = −0.28). However, there was no significant interaction between time and BPA exposure group.

**Table 3:** Comparison of sex hormone levels and metabolic indices at different time points according to urinary bisphenol A tertile.

	Time	Total	BPA			*p*
			Tertile 1 (n = 17)	Tertile 2 (n = 16)	Tertile 3 (n = 15)	
BMI (kg/m^2^)	T1	16.16 (2.31)	15.79 (2.15)	15.92 (1.96)	16.82 (2.81)	0.41
	T2	16.26 (2.02)	15.66 (1.34)	16.32 (2.19)	16.89 (2.37)	0.23
LH (IU/L) ^‡^	T1	0.57 (0.25–0.93)	0.57 (0.24–0.86)	0.57 (0.27–0.66)	0.75 (0.33–1.43)	0.58
	T2	0.75 (0.24–1.60)	0.75 (0.24–2.26)	0.47 (0.24–1.70)	0.94 (0.24–1.51)	0.89
Estradiol (pg/mL) ^‡^	T1	3.75 (2.50–10.30)	2.50 (2.50–7.05)	2.50 (2.50–9.30)	10.35 (2.50–13.30)	0.05
	T2	7.10 (2.50–13.80)	3.95 (2.50–7.15)	6.95 (2.50–25.05)	11.60 (7.10–17.80)	0.02
Androstenedione (ng/mL) ^‡^	T1	0.55 (0.37–0.82)	0.45 (0.32–0.64)	0.68 (0.42–0.86)	0.63 (0.42–0.87)	0.22
	T2	0.51 (0.37–0.80)	0.38 (0.34–0.53)	0.59 (0.43–0.83)	0.66 (0.41–1.19)	0.01
DHEA (ng/mL) ^‡^	T1	0.61 (0.05–1.05)	0.40 (0.05–0.76)	0.61 (0.10–0.90)	0.85 (0.06–1.39)	0.32
	T2	0.65 (0.09–1.48)	0.56 (0.05–0.77)	0.60 (0.09–1.87)	1.37 (0.23–1.88)	0.07
Testosterone (ng/mL) ^‡^	T1	0.05 (0.04–0.07)	0.04 (0.03–0.06)	0.05 (0.04–0.09)	0.06 (0.04–0.09)	0.33
	T2	0.09 (0.08–0.15)	0.09 (0.06–0.11)	0.09 (0.08–0.19)	0.13 (0.08–0.20)	0.09
Insulin (μIU/mL)	T1	7.73 (1.96)	7.02 (1.44)	8.03 (2.23)	8.23 (2.07)	0.17
	T2	8.33 (2.10)	7.25 (1.87)	8.95 (2.46) ^†^	8.94 (1.35)	0.02
Glucose (mmol/L)	T1	4.26 (0.32)	4.23 (0.26)	4.45 (0.30)	4.20 (0.36)	0.05
	T2	4.51 (0.38)	4.41 (0.41)	4.64 (0.34)	4.49 (0.37)	0.21
HOMA-IR	T1	1.49 (0.43)	1.32 (0.30)	1.60 (0.49)	1.55 (0.46)	0.15
	T2	1.69 (0.50)	1.42 (0.44)	1.85 (0.55) ^†^	1.81 (0.38)	0.02

Results are presented as means plus standard deviation. Results for right skewed data are presented as medians (interquartile range). LH, luteinizing hormone; DHEA, dehydroepiandrosterone; HOMA-IR, homeostasis model assessment of insulin resistance. HOMA-IR was calculated as (plasma glucose (mmol/L) × insulin (μIU/mL))/22.5. ^†^ Tukey-adjusted *p* < 0.05 for multiple comparisons (reference: tertile 1). ^‡^ *p* value by Kruskal-Wallis test.

**Table 4:** Repeated measures multivariate analysis of variance for urinary bisphenol A tertiles after 1-year of follow-up.

	Time	Urinary BPA tertiles			*p* value		
		Tertile 1 (n = 17)	Tertile 2 (n = 16)	Tertile 3 (n = 15)	*p1*	*p2*	*p3*
BMI (kg/m^2^)	T1	16.33 (15.21–17.45)	15.47 (14.34–16.60)	16.62 (15.44–17.81)	0.35	0.48	0.43
	T2	16.18 (15.20–17.16)	15.99 (15.00–16.98)	16.72 (15.69–17.76)	0.57		
LH (IU/L)	T1	0.67 (0.46–0.98)	0.53 (0.37–0.78)	0.65 (0.44–0.97)	0.65	0.81	0.85
	T2	0.73 (0.47–1.14)	0.74 (0.48–1.15)	0.71 (0.45–1.12)	0.99		
Estradiol (pg/mL)	T1	5.68 (4.20–7.69)	9.08 (6.63–12.43)	9.05 (6.53–12.54)	0.06	0.62	0.01
	T2	6.28 (4.32–9.14)	12.20 (8.26–18.03)	12.87 (8.59–19.29) ^†^	0.02		
Androstenedione (ng/mL)	T1	0.40 (0.30–0.52)	0.65 (0.50–0.85) ^†^	0.62 (0.47–0.82)	0.02	0.20	0.01
	T2	0.30 (0.22–0.41)	0.64 (0.47–0.89) ^†^	0.65 (0.46–0.90) ^†^	0.01		
DHEA (ng/mL)	T1	0.19 (0.09–0.42)	0.35 (0.16–0.76)	0.52 (0.24–1.18)	0.20	0.61	0.07
	T2	0.22 (0.10–0.51)	0.37 (0.16–0.87)	0.93 (0.39–2.22)	0.07		
Testosterone (ng/mL)	T1	0.04 (0.03–0.06)	0.05 (0.04–0.08)	0.07 (0.05–0.10)	0.26	0.96	0.12
	T2	0.08 (0.06–0.11)	0.10 (0.08–0.14)	0.13 (0.10–0.16)	0.08		
Insulin (μIU/mL)	T1	7.95 (6.69–9.21)	8.75 (7.39–10.10)	8.87 (7.59–10.14)	0.38	0.86	0.10
	T2	7.60 (6.30–8.89)	8.87 (7.48–10.27)	8.88 (7.58–10.19)	0.14		
Glucose (mmol/L)	T1	4.23 (4.04–4.43)	4.40 (4.19–4.60)	4.15 (3.96–4.35)	0.09	0.43	0.19
	T2	4.51 (4.26–4.75)	4.68 (4.42–4.93)	4.59 (4.34–4.83)	0.48		
HOMA-IR	T1	1.51 (1.22–1.79)	1.72 (1.42–2.02)	1.66 (1.37–1.94)	0.40	0.76	0.09
	T2	1.51 (1.21–1.82)	1.83 (1.51–2.15)	1.82 (1.51–2.12)	0.12		

Results are presented as least squares means (LSmeans) with 95% confidence intervals for repeated-measures MANOVA with adjustments for age, household income, urinary creatinine level, and pubertal level (as measured by Tanner stage at baseline) and additionally adjusted for obesity (normal or overweight) in insulin, glucose, and HOMA-IR. LH, luteinizing hormone; DHEA, dehydroepiandrosterone; HOMA-IR, homeostasis model assessment of insulin resistance. HOMA-IR was calculated as (plasma glucose (mmol/L) × insulin (μIU/mL))/22.5. *p1*: *p* value for group difference at each time point. *p2*: *p* value for interaction between time and group. *p3*: *p* value for group difference for repeated measures between subjects. Significance was derived from multiple comparisons using the Tukey-adjusted method. ^†^ *p* < 0.05 compared to tertile 1.

## 4. Discussion

Through our study, we found that exposure to relatively high levels of BPA increased androgenic hormone levels in preadolescent aged girls, and the adverse effect of BPA on endocrine metabolism appeared to persistent. Secondly, BPA may also potentially contribute to the development of insulin resistance, although this study showed marginal significance. Moreover, our results support several possible explanations and suggest the need for further studies with an increased scope in terms of both period of observation and size.

PCOS, a common complex endocrinopathy in women of reproductive age, is one of the leading causes of infertility [17]. Although its etiology is unknown, some studies have suggested that excessive androgen and insulin levels may be key endocrine players in the pathogenesis of PCOS [18]. Insulin acts synergistically to enhance signals to produce androgen hormones and can also maintain androgen production by interfering with hepatic sex hormone-binding globulin (SHBG) production, which in turn increases the likelihood of insulin increase [19]. This snowball effect results in worsening PCOS symptoms. In the present study, insulin concentration at baseline was positively correlated with later levels of androstenedione (*r* = 0.36, *p* = 0.02) and testosterone (*r* = 0.37, *p* = 0.01), after adjustment for BMI, age, and pubertal level (measured by Tanner stage) at baseline (data not shown). Conversely, androgen concentrations at baseline also showed positive correlations with subsequent insulin levels, but androstenedione only showed marginal significance (*r* = 0.36, *p* = 0.02 for testosterone and *r* = 0.28, *p* = 0.07 for androstenedione). In women, androgen hormones are normally produced by the ovaries and the adrenal glands [20], but effects of excess levels of androgen on health are unclear. Nevertheless, several lines of evidence suggest that elevated androgen levels have negative effects on the reproductive system [21]. Hyperandrogenemia may adversely affect the normal negative feedback effects of estradiol and progesterone on gonadotropin-releasing hormone (GnRH) secretion and may induce follicular atresia [21], which may lead to reduced fertility.

Having too much androgen may be caused by a genetic condition [22] or fat mass [23]. It was recently suggested that endocrine disruptor compounds may be stimulators, especially BPA [24]. One *in vitro* study provided evidence that BPA exposure leads to elevated testosterone synthesis in rat ovarian theca-interstitial cells [25]. BPA also indirectly increases testosterone concentrations by inhibiting testosterone catabolism [26]. Lee *et al*. proposed that BPA partially disrupts the binding of androgens to the androgen receptor (AR), resulting in elevated free androgen levels [3], but not all [27]. Meanwhile, Takeuchi *et al*. suggested that BPA is maintained at high levels because androgens interfere with the activity of BPA clearance enzymes [9]. In addition, a growing body of evidence indicates that the female gonad appears to be a particularly sensitive target of BPA disruption, and that BPA impairs follicle growth and induces ovarian abnormalities [28]. In accordance with the suggested mechanism, androgenic hormones and insulin mostly showed high levels in girls in the top BPA tertile in the present study.

Although evidence from *in vivo* and *in vitro* studies has suggested several biological mechanisms of BPA activity, all of them remain uncertain. Moreover, there is still little information on human exposure to BPA. Recently, two case-control studies found that women with PCOS had higher androgen and BPA levels than women without PCOS [7,24]. In children, there have been no reports except for an abstract from a meeting describing negative correlations of BPA with androstenedione (*ρ* = −0.17, *p* = 0.16) and testosterone (*ρ* = −0.26, *p* = 0.03) [29]. Unlike the study mentioned above, the results of our study support the suggested mechanism of BPA.

In addition, a previous animal study suggested that prenatal BPA exposure has an important irreversible effect on the development of disease susceptibility. As some of the subjects had maternal BPA concentrations determined during pregnancy, we analyzed the Spearman’s correlation between BPA during pregnancy and current BPA concentration. A positive relationship was observed, but it was not significant (*ρ* = 0.10, *p* = 0.63). This result is similar to that of a previous study [30]. Thus, we believe that the effect of prenatal BPA exposure may be independent of current BPA exposure. There is a need to study the effect of BPA exposure further during this critical period.

In the present study, the median urinary BPA concentration determined by HPLC/FD was 12.0 ng/mL (9.1 μg/g creatinine). Many studies from several different countries have examined BPA in adults, adolescents, and children. In studies of children aged 6–11 years, the urinary BPA concentration varied from 2.0 to 4.3 ng/mL [16]. Yang *et al*., who studied Koreans, reported that the median urinary BPA concentration was 14.9 μg/g creatinine in 172 middle-aged adults [31] and 4.6 μg/g creatinine in 26 children with an average age of 8.7 years [32]. BPA concentrations can differ for systemic reasons, such as differences in sample collection, measurement methods, sample storage, and control for contamination, as well as age, race, and sex [16]. These various factors may account for the variation in BPA concentration.

Although this pilot study derived some meaningful findings, it had several limitations. First, urinary BPA concentration was measured only once in a small sample size, which may have reduced precision. The uncertainty of spot urine measurement has been suggested, but reproducibility is moderate within individuals for up to several months [33,34,35]. The correlation for 48 subjects at two time points was moderate (*ρ* = 0.36, *p* < 0.02), which is similar to a previous report [35]. In addition, Calafat *et al*. suggested that the total BPA urinary concentration is a valid biomarker of BPA exposure [13].

In our study, the limit of detection (LOD) value (2.0 ng/mL) is higher than reported previously. For that reason, the median value of BPA seems higher in the present study. We repeated measurements in samples with higher concentrations for increased accuracy. The measurements consistently showed high concentrations. We believe our results to be reliable even though the BPA concentration was high; however, the accuracy was limited by the experimental design. Finally, it may be inappropriate to generalize the results of this study due to the lack of representation of girls of pre-reproductive ages. However, selection bias seems to be minor according to the lack of differences in baseline characteristics between girls who fully attended follow-up and those who attended only once.

Nevertheless, this is the first study to prospectively investigate the association between BPA exposure and susceptibility related to PCOS in preadolescent girls, and our results will increase understanding of BPA as an endocrine disrupter that potentially leads to elevation of androgenic hormone levels. The limited studies in the literature were mostly conducted using cross-sectional designs and adult populations. In general, children are likely to be more vulnerable to exposure of BPA than adults [16]. In addition, puberty is a dynamic period of hormonal and body size changes. Thus, further study is needed to determine whether pre-pubertal BPA exposure accelerates the increases in androgenic hormone levels in girls of reproductive ages.

Although no difference was observed between the changes in sex or metabolic hormones over time between the BPA groups, increased exposure to BPA was related to higher levels of estradiol and androstenedione at both baseline and follow-up. Furthermore, humans can be simultaneously exposed to other environmental toxicants including phthalates as well as BPA and the potential combination may lead to adverse effects [36]. Trasande *et al*. [37] reported an additive effect between phthalates and high BPA exposure (highest quartile) on insulin resistance in adolescents. 

## 5. Conclusions

Thus, our results provide a basis for further studies with larger sample sizes and longer follow-up to identify the causal relationship and mechanisms linking BPA and other EDCs with the regulation of endocrine processes.
